# Progression of keratoconus in the contralateral eye following acute corneal hydrops: A retrospective study

**DOI:** 10.1371/journal.pone.0324750

**Published:** 2025-05-22

**Authors:** James Steven Lewis, Charles N.J. McGhee, Jay Meyer

**Affiliations:** University of Auckland, Faculty of Medical and Health Sciences - Ophthalmology, Auckland, New Zealand; University of Warmia, POLAND

## Abstract

Keratoconus is a progressive eye disease characterised by thinning and bulging of the cornea, leading to distorted vision. Acute corneal hydrops, a rare and severe complication of keratoconus, occurs when fluid accumulates in the cornea due to a sudden break in its innermost layer, often causing pain, significant visual impairment, and sometimes requiring corneal transplantation. To better understand the risk of disease progression in the contralateral (unaffected) eye, we conducted a retrospective study at two major tertiary hospitals in Auckland, New Zealand. The study included 45 eyes from 45 patients presenting with acute corneal hydrops. Inclusion and exclusion criteria were rigorously applied to ensure that only patients eligible for corneal collagen crosslinking (CXL) at baseline were included, enhancing the clinical relevance of the findings. Patients were followed for a mean duration of 4.73 years. The results showed that 67% of contralateral eyes exhibited progression in at least one tomographic parameter, with 53.3% demonstrating progression in both anterior curvature and corneal thickness parameters. Higher baseline anterior curvature values and male sex were significantly associated with an increased risk of progression (p = 0.022 and p = 0.046, respectively). Nearly half of the eyes with progression had a thinnest corneal thickness of ≥400μm, making them eligible for CXL at initial presentation. However, 36% of these eligible eyes dropped below this threshold over the follow-up period, underscoring the importance of timely intervention to preserve treatment options. These findings highlight the importance of regular monitoring of the contralateral eye following acute corneal hydrops, particularly in male patients or those with advanced baseline disease. Immediate CXL may be considered in select cases to prevent disease progression. This study also emphasises the need for early intervention strategies and targeted healthcare services for populations at higher risk, including Māori and Pacific Peoples in Aotearoa-New Zealand, who experience disproportionately high burdens of keratoconus and health inequities.

## Introduction

Keratoconus is a progressive corneal ectasia that produces thinning and conical protrusion of the cornea. While considered a bilateral condition, it is frequently asymmetric in severity and typically results in myopia and progressive irregular astigmatism of the affected eyes [1]. Potential sequelae include corneal scarring, corneal hydrops, and, ultimately, loss of best-corrected visual acuity.

Keratoconus typically manifests during puberty and may progress into the third or fourth decade of life when limited progression or stabilization usually occurs [2]. Without treatment to alter progression of the disease, approximately 20% of eyes may require corneal transplantation to provide visual rehabilitation [3].

Studies have been conducted on the risk factors for the development and progression of keratoconus [4,5]. However, the risk of progression of keratoconus in the contralateral eye following an episode of acute corneal hydrops has not been characterised. It is important for clinicians to know the risk of progression to appropriately risk-stratify patients when determining whether to proceed with immediate corneal collagen crosslinking (CXL) in the contralateral eye or to first observe for signs of clinical or tomographic progression before performing CXL. To date, the magnitude of the risk of disease progression has not been determined.

This study aimed to investigate the rate of tomographic progression of keratoconus in the contralateral eye in a series of patients presenting with acute corneal hydrops at two major tertiary centres for keratoconus in Auckland, New Zealand.

## Subjects and methods

### Design

This was a retrospective cohort study of individuals with keratoconus who presented with acute corneal hydrops at the Department of Ophthalmology, Auckland District Health Board, or Counties Manukau District Health Board, Auckland, New Zealand, between January 2010 and July 2021. This study was approved by the Auckland Health Research Ethics Committee (#3195) and performed in accordance with the Declaration of Helsinki. Due to the retrospective nature of the study, the requirement for informed consent from participants was waived.

The data for this study were accessed on 26 November 2022. During data collection, authors had access to identifying information, such as National Health Index (NHI) numbers, necessary for accurate data collation and analysis. This information was securely stored on a password-protected spreadsheet within the hospital servers, adhering to institutional security policies. Post-collection, the data were anonymised prior to analysis.

The original identifiable data remain securely stored on the hospital system. Given the involvement of minors in the study, this data will be retained until participants reach the age of 16 years plus an additional 10 years.

### Inclusion and exclusion criteria

The inclusion criteria were diagnosis of keratoconus, examination during an episode of acute corneal hydrops, at least 6 months of follow-up, and two tomography scans performed at least 6 months apart using the same tomographer. Keratoconus was diagnosed by a corneal specialist based on a clinical examination combined with computerized tomography, slit-lamp biomicroscopy, and refraction.

The exclusion criteria included prior trauma or corneal surgery, corneal scarring in the contralateral eye (including previous corneal hydrops), contact lens wear within 2 weeks of corneal tomography, CXL performed in the contralateral eye prior to documentation of keratoconus progression, or a diagnosis of Down syndrome (trisomy 21). Patients with Down syndrome (trisomy 21) were excluded because keratoconus in these individuals often follows a distinct, more aggressive course, with earlier onset and rapid progression [6–8]. These differences in disease behaviour and management considerations warrant separate analysis, as including them could have confounded our study’s findings.

### Examinations

All patients were examined clinically and underwent corneal tomography using either the Galilei dual Scheimpflug system (Ziemer Ophthalmic Systems AG, Port, Switzerland) or Pentacam HR (Oculus Optikgeraete GmbH; Wetzlar, Germany). Measurements were conducted by experienced ophthalmic technicians using the manufacturer’s recommended acquisition protocol. The devices were serviced and calibrated regularly, according to the manufacturer’s specifications.

### Analysis

Demographic data, follow-up period, and documented history of asthma, eczema, or hay fever were also collected. All patients were advised to avoid eye rubbing and medical management was offered to patients with atopic conditions. The self-reported ethnicities were recorded.

The following parameters were assessed: flat simulated keratometry of the anterior surface (K1A), steep simulated keratometry of the anterior surface (K2A), central corneal thickness (CCT) and thinnest corneal thickness (TCT). These parameters were compared between the baseline and final reviews for each eye.

Data to determine the thresholds of repeatability of the tomographic parameters used in this study on keratoconic eyes were obtained from previous work by our group [9]. Tomographic progression was defined as an increase in either of the anterior curvature parameters or a decrease in either pachymetric parameter beyond the repeatability limits. Test–retest variability thresholds with one sided 95% limits were calculated as 2.33 (1.645 × √2) times the within-subject intrasession standard deviation. The test–retest variability thresholds (minimum change required to be considered progression) for each tomographer were calculated as follows:

Galilei dual Scheimpflug system

K1A + 1.40 dioptres (D), K2A + 1.50 D, CCT −8.40μm, TCT −11.10μm.

Pentacam HR

K1A + 0.70 D, K2A + 0.80 D, CCT −11.3μm, TCT −23.6μm.

Data management was performed using Microsoft Excel for Mac version 16.68 (Microsoft Corporation, Redmond, Washington, USA), and statistical analyses were performed using IBM SPSS Statistics for Mac V.29.0.0.0 (241) (International Business Machines Corporation, Armonk, New York, USA). Data are reported as the mean ± SD. The univariate analyses consisted of Student’s t-test (two-tailed), which was used to compare means between groups for continuous variables. The chi-square test with Bonferroni correction was performed to assess differences between categorical variables [10]. A *p* value of <0.05 was considered significant.

## Results

A total of 248 eyes from 228 patients with acute corneal hydrops during the study period were evaluated for inclusion in the study. Of these, 45 eyes from 45 patients were included in the analysis of keratoconus progression in the contralateral (non-hydrops) eye. The mean age of studied patients was 25.9 ± 9.0 (range, 9.4–50.6) years of which 55.6% were male and the mean follow-up period was 4.7 ± 3.1 (range, 0.5–12.3) years. The self-reported ethnicities included Asian (2.2%), European (6.7%), Māori (24.4%), and Pacific people (66.7%). Of the included patients, 73.3% had a documented history of atopy (asthma, eczema, or allergic rhinitis), and 40% had a documented history of eye-rubbing.

Reasons for exclusion included corneal scarring in the contralateral (non-hydrops) eye, including prior corneal hydrops in the contralateral eye (52 eyes, 25.6%), less than 6 months follow-up (47 eyes, 23.2%), lack of follow-up tomography due to inability to obtain a reliable scan or because a different tomographer was used (36 eyes, 17.7%), lack of initial tomography during the acute episode of hydrops (16 eyes, 7.9%), corneal graft in the contralateral eye (16 eyes, 7.9%), CXL in the contralateral eye after the episode of hydrops but prior to evidence of progression (12 eyes, 5.9%), less than 6 months between valid scans (14 eyes, 6.9%), Down syndrome (8 eyes, 3.9%), and 2 eyes (1.0%) were excluded due to previous CXL in the contralateral (non-hydrops) eye prior to the hydrops episode.

At the time of initial tomography, 18 (40.0%) contralateral eyes had Amsler-Krumeich (AK) stage I keratoconus, 7 (15.6%) were AK stage II, 6 (13.3%) were AK stage III, and 14 (31.1%) were AK stage IV [11].

There was a significant difference in K1A, K2A, and TCT when comparing overall baseline and final mean measurements (Table 1).

**Table 1:** Mean tomographic values at initial and final examination.

Parameter	Initial exam	Final exam	Mean change ± SD (95% CI)	*p* value
K1A (D)	50.0 ± 8.0	52.1 ± 9.5	2.0 ± 5.2 (0.5 to 3.6)	**<0.001**
K2A (D)	54.4 ± 8.9	57.0 ± 10.4	2.6 ± 4.9 (1.1 to 4.1)	**<0.001**
CCT (μm)	434.1 ± 81.5	419.7 ± 87.8	-14.4 ± 78.4 (-37.9 to 9.1)	.2
TCT (μm)	401.6 ± 83.5	369.1 ± 110.1	-32.6 ± 84.3 (-57.9 to -7.2)	**<0.001**

**Table 1 Legend:** Mean tomographic values at initial and final examination for 45 eyes, after a mean follow-up time of 4.73 ± 3.09 years. Results at initial and final examination are shown as the mean ± standard deviation, with the mean change including the 95% confidence interval of the difference in values between the examinations. The statistical significance is shown, with bold values denoting significance at the p < 0.05 level.

Abbreviations: *SD* standard deviation *D* diopters, *CI* confidence interval, *K1A* flat simulated keratometry of the anterior surface, *K2A* steep simulated keratometry of the anterior surface, *CCT* central corneal pachymetry, *TCT* thinnest corneal pachymetry.

The overall rate of tomographic progression in contralateral eyes following an episode of acute corneal hydrops was 67% (30/45 eyes), based on changes in at least one tomographic parameter beyond the repeatability limits.

If a stricter definition of progression was used based on an increase in an anterior curvature parameter (K1A and/or K2A) and a decrease in a pachymetric parameter (CCT and/or TCT) beyond the repeatability limits, the rate of progression was 53.3% (24/45 eyes).

Of the 30 eyes that demonstrated tomographic progression in at least one parameter, 14 had a TCT ≥ 400µm, indicating that approximately half would have been eligible for CXL using the traditional cut-off of 400µm. Of these 14, there were five (36%) that were no longer above the 400µm cut-off after the follow-up period.

On univariate analysis, a higher K2A value was associated with an increased risk of KC progression in these eyes (*p* = 0.022, Table 2). On multivariate analysis, male sex was associated with an increased risk of KC progression (*p* = 0.046, Table 2).

**Table 2:** Variables at initial examination for eyes that did and did not demonstrate tomographic progression.

Variable		Progression		Univariate	Multivariate
		Yes (*n* = 30)	No (*n* = 15)	*p* value	*p* value
Sex	Male	18 (72.0%)	7 (28.0%)	0.396	**0.046**
	Female	12 (60.0%)	8 (40.0%)		
Ethnicity	Māori	6 (54.5%)	5 (45.5%)	0.344	0.285 0.201 0.410 1.00
	Pacific Peoples	22 (73.3%)	8 (26.7%)		
	European	1 (33.3%)	2 (66.7%)		
	Asian	1 (100%)	0 (0.0%)		
Atopy	Yes	24 (72.7%)	9 (27.3%)	0.153	0.908
	No	6 (50.0%)	6 (50.0%)		
Documented eye rubber	Yes	13 (72.2%)	5 (27.8%)	0.519	0.894
	No	17 (63.0%)	10 (37.0%)		
Age at time of hydrops (years)		24.5 ± 7.7	28.6 ± 11.0	0.208	0.314
Follow-up (years)		5.1 ± 3.2	3.9 ± 2.9	0.216	0.489
K1A (D)		51.4 ± 8.9	47.3 ± 5.1	0.061	0.626
K2A (D)		56.2 ± 9.8	50.8 ± 5.5	**0.022**	0.100
CCT (μm)		424.5 ± 81.5	453.2 ± 80.9	0.273	0.293
TCT (μm)		390.4 ± 82.2	424.2 ± 84.2	0.211	0.989

Table 2 Legend: Variables at initial examination for eyes which did and did not demonstrate tomographic progression. For categorical variables, results are numbers of eyes. For continuous variables, results are shown as the mean ± standard deviation. For univariate and multivariate analyses, the statistical significance is shown, with bold values denoting significance at the p < 0.05 level.

*n* number, *Atopy* Documented diagnosis of asthma, eczema or hay-fever, *D* diopters, *K1A* flat simulated keratometry of the anterior surface, *K2A* steep simulated keratometry of the anterior surface, *CCT* central corneal pachymetry, *TCT* thinnest corneal pachymetry.

## Discussion

To date, a variety of risk factors have been identified to be associated with keratoconus, including ethnicity, climate, geography, and external factors such as eye-rubbing and atopy [12–15]. Studies have also identified that those patients with a younger age of presentation and more advanced disease at presentation are most at risk of further disease progression [16–18]. These risk factors are summarised in Table 3. Patients with advanced keratoconus in at least one eye are also at higher risk for progression in both eyes [16]. Acute corneal hydrops is an infrequent but significant complication of keratoconus and has been reported to occur in approximately 2.6% to 2.8% of patients [19]. While studies have confirmed that the risk factors for acute corneal hydrops are similar to those for the development of keratoconus [20], there is a lack of research on whether an episode of acute corneal hydrops in one eye is associated with keratoconus progression in the contralateral eye.

**Table 3:** Risk factors for keratoconus and progression of disease.

Risk Factor	Description
Genetic predisposition	Family history of keratoconus increases risk [1].
Ethnicity	Higher prevalence in Māori, Pacific Peoples, Middle Eastern, and South Asian populations [21].
Eye rubbing	Strongly linked to progression [14].
Atopy (asthma, eczema, allergies)	Increases risk and severity [13].
Age of onset	Usually presents during puberty, progresses into the 30s. Age < 19 at presentation linked to up to 88% risk of progression [5,22].
Trisomy 21 (Down syndrome)	High prevalence, aggressive progression [6–8].

Keratoconus is generally considered a bilateral condition [23], and the present study provides updated evidence on contralateral progression in the specific context of acute corneal hydrops. This study uniquely focuses on patients who already have hydrops in one eye, a high-risk scenario that was not analysed in prior literature. Furthermore, modern tomographic progression criteria have been applied and eligibility considered, as such parameters were not applicable in studies prior to the development of the CXL procedure. These additions contribute new clinical insights for contemporary keratoconus management.

The present study identified a significant rate of progression of keratoconus in the contralateral eye following the development of acute corneal hydrops, with two-thirds demonstrating evidence of progression of keratoconus in at least one tomographic parameter and approximately half with progression of two parameters. Knowledge regarding this risk of progression is valuable for clinicians to aid in decision making and patient counselling regarding potential interventions for the non-hydrops eye, such as whether to intervene with CXL immediately or whether to wait for documented progression before performing CXL.

CXL for the treatment of progressive keratoconus has been performed and refined since its initial implementation in 2003 by Wollensack et al [24] into the widely used Dresden protocol. Numerous studies have validated its efficacy at halting the progression of keratoconus in follow-up periods as long as 10 years, as well as an excellent safety profile [12,24–27]. Health economic data indicate that timely intervention with CXL can reduce the need for penetrating keratoplasty by up to half [28,29]. In addition, research has confirmed that CXL is cost-effective compared to conventional management with penetrating keratoplasty [30–32], with one study reporting that patients who underwent CXL spent, on average, 27.9 fewer years in advanced disease states, had 1.88 additional quality-adjusted life-years, and lower direct treatment costs [33]. Considering the impact of reduced productivity loss, the same study found that CXL was associated with a lifetime cost-savings of $43,759 per patient [33]. While many contralateral eyes in our study had advanced keratoconus at initial presentation, treatment decisions were guided by standard clinical practice at the time, which typically required documented progression before initiating CXL. Additionally, some eyes had borderline or sub-threshold corneal thickness, limiting the feasibility of immediate intervention. This finding suggests that a more proactive treatment strategy could be warranted in select high-risk cases to prevent loss of eligibility for CXL over time.

Of the eyes that demonstrated tomographic progression, nearly half had a TCT of ≥400µm. *Prima facie*, these eyes would have been eligible for CXL under the Dresden protocol, accruing benefits to the patient, society, and healthcare sector outlined above.

However, over a third of the eyes that would have been eligible for immediate CXL in the contralateral eye were no longer eligible after the follow-up period based on a cut-off of 400µm. Thus, immediate CXL for the contralateral eyes may be warranted for select eyes, as the window of opportunity for intervention may be limited in eyes with borderline or low corneal thickness, and further progression may further reduce the thickness and limit the ability to perform CXL. This study also found that a high proportion of patients did not have adequate follow-up to allow for determination of progression due to clinic non-attendance.

The high rate of progression and risk of loss to follow-up are also factors that suggest that it would be reasonable to consider immediate CXL in the contralateral eye without waiting for evidence of progression in select eyes, provided that there are no contraindications to the procedure. In addition, the risks of the CXL procedure must be considered as well and it may be prudent to wait at least until the resolution of the acute corneal hydrops before performing CXL since the procedure will further reduce the vision during postoperative healing period.

Key clinical factors which might prompt a decision to perform CXL in the fellow eye after an episode of acute corneal hydrops might include a younger age at presentation, as well as evidence of advanced keratoconus at the time of presentation (TCT 400–450um). This is in line with studies which show that 88% of paediatric keratoconic patients progressed while being observed [22], and our data which showed that one third of patients became ineligible for CXL while being observed for progression.

Another important factor in this decision is a judgement on the likelihood of the patient being ‘lost to follow-up’, as happened with a significant proportion of patients in our study (23.2%). This may happen in patients with low healthcare engagement, socioeconomic barriers to healthcare access, or other systemic conditions preventing routine monitoring.

The patient’s opinion should be sought and given weight. From their perspective, losing vision in one eye due to hydrops could be traumatising, and treatment without evidence of progression may give them important reassurance that everything is being done to preserve vision in their fellow eye before further damage occurs. Conversely, given the pain and visual disability associated with acute hydrops and the small risks associated with the CXL procedure, they may prefer to wait for evidence of documented progression. If CXL without evidence of documented progression is to occur, a shared decision should be reached between patients, family, and the treating clinician to ensure that timely intervention is balanced with resolution of acute pain and visual disability from the episode of acute hydrops.

All these considerations must be balanced with the broader impact on the delivery of CXL for those patients already awaiting a procedure to ensure fairness. One study noted that treatment of all high-risk eyes in their cohort may have avoided 140 unnecessary clinic visits, and saved an estimated £18,900 in clinic costs and a mean of 0.79 lines of vision, meaning that this approach may be both medically and economically justified [34].

In the context of Aotearoa-New Zealand, there is an ethnic disparity in the burden of keratoconus, with the disease being 2–4 times more prevalent in patients of Māori and Pacific Peoples ethnicity [15,21]. As patients from these ethnicities are known to suffer health inequities and barriers to access health care, this finding has important implications for primary screening and the planning and delivery of health services locally [35,36].

This study had several limitations, including a high proportion of individuals lost to follow-up and a significant number of patients for whom we were unable to obtain tomographic data. This was partly because one of the tomography machines utilized at our centre was withdrawn from the service and replaced with the current model. This study also included data from both Galilei and Pentacam tomographers. While the same tomographer was used sequentially for each patient to evaluate progression, differences between the two tomographers limited the extent to which tomographic parameters could be compared between patients in this study. Finally, our study also had strict inclusion and exclusion criteria based on whether patients who progressed would have been eligible for CXL at the time of their first tomographic scan, such as the exclusion of eyes with corneal scarring. This ultimately decreased the number of patients who could be assessed for tomographic progression.

In summary, these data were drawn from a very large series of patients who presented with an uncommon complication of keratoconus, acute corneal hydrops, over an extended period at two major tertiary ophthalmology centres. They provide evidence that acute corneal hydrops is associated with the tomographic progression of keratoconus in the contralateral eye. Given the excellent safety profile associated with CXL, its known efficacy, the reduction in morbidity associated with the treatment of progressive keratoconus, and the potential for cost savings, clinicians closely monitor the contralateral eye following corneal hydrops closely and consider CXL when deemed clinically indicated.
